# A review of the regulatory mechanisms of extracellular vesicles-mediated intercellular communication

**DOI:** 10.1186/s12964-023-01103-6

**Published:** 2023-04-13

**Authors:** Ya-Juan Liu, Cheng Wang

**Affiliations:** 1 Key Laboratory of Molecular Target and Clinical Pharmacology, The NMPA and State Key Laboratory of Respiratory Disease, School of Pharmaceutical Sciences and the Fifth Affiliated Hospital, Guangzhou Medical University, Guangzhou, 511436 China; 2 Smurfit Institute of Genetics, Trinity College Dublin, Dublin, D02 VF25 Ireland

**Keywords:** Extracellular vesicle, Exosome, Intercellular communication, EV uptake, EV biogenesis

## Abstract

Extracellular vesicles (EVs) are small, membrane-bound structures that are released from cells into the surrounding environment. These structures can be categorized as exosomes, microvesicles, or apoptotic vesicles, and they play an essential role in intercellular communication. These vesicles are attracting significant clinical interest as they offer the potential for drug delivery, disease diagnosis, and therapeutic intervention. To fully understand the regulation of intercellular communication through EVs, it is essential to investigate the underlying mechanisms. This review aims to provide a summary of the current knowledge on the intercellular communications involved in EV targeting, binding, and uptake, as well as the factors that influence these interactions. These factors include the properties of the EVs, the cellular environment, and the recipient cell. As the field of EV-related intercellular communication continues to expand and techniques improve, we can expect to uncover more information about this complex area, despite the current limitations in our knowledge.

**Supplementary Information:**

The online version contains supplementary material available at 10.1186/s12964-023-01103-6.

## Background

**Fig. 1:**
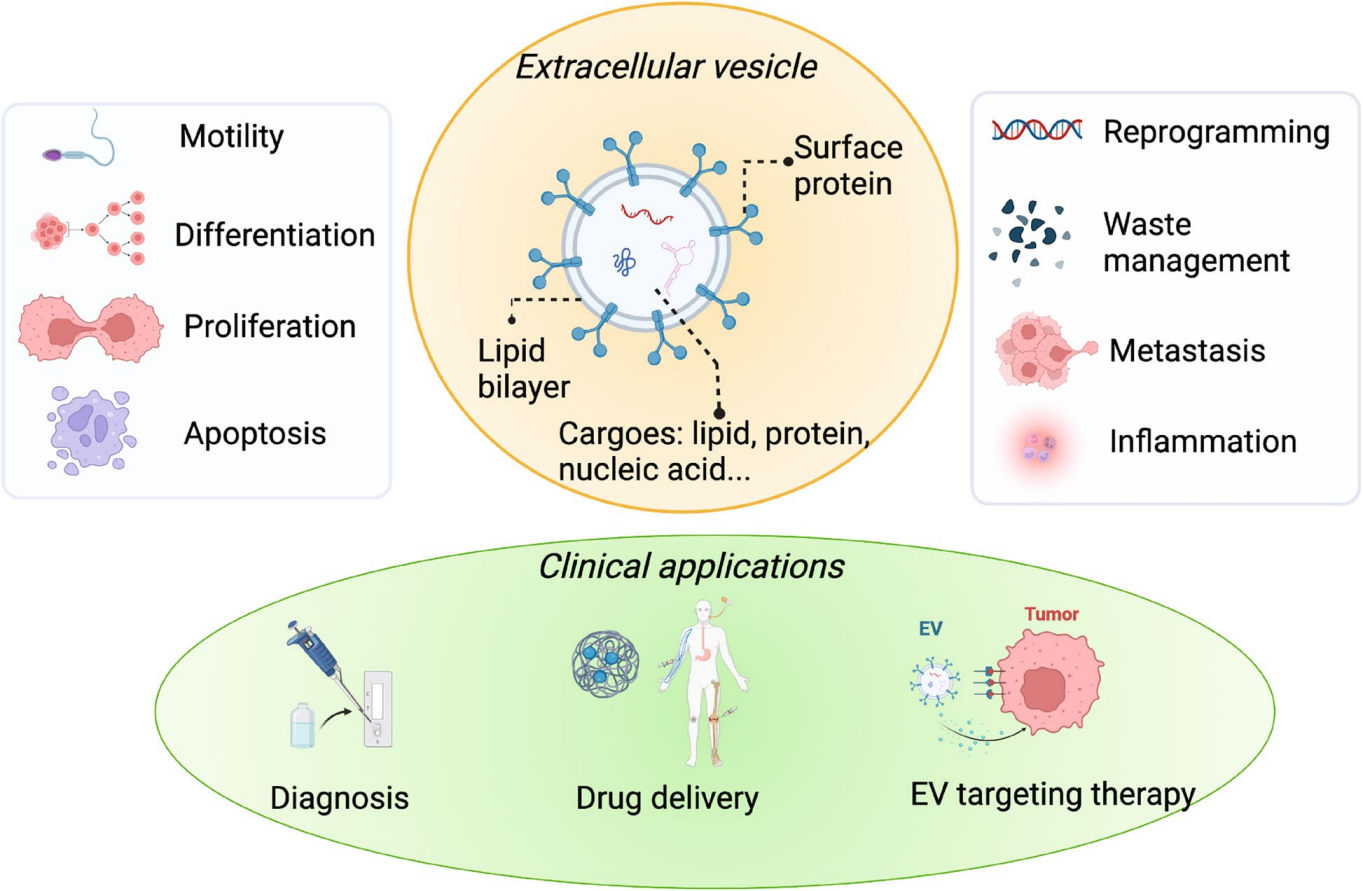
The general introductions of EVs, including the compositions, the related physiological and pathological processes, and their clinical applications. EVs are lipid membranous vesicles. The communication based on EVs is related to their surface proteins and their cargoes including protein, lipid, and nucleic acids. EVs participate in diverse biological processes, such as cell motility, differentiation, proliferation, apoptosis, reprogramming, waste management, metastasis, and inflammation. The biological processes related to EVs are linked with their clinical potentials such as diagnosis based on their biomarker properties, drug delivery with their targeting properties, and targeting therapy using their communication properties

Extracellular vesicles (EVs) play a critical role in mediating and regulating intercellular communication associated with both physiological and pathological processes [1–4]. EVs (Fig. 1) are lipid membranous vesicles consisting of proteins, lipids, and nucleic acids, which are heterogenous associated with the composition and function [5]. EVs can be classified into several types based on their origin, such as exosomes, microvesicles, and apoptotic vesicles [2, 5]. Exosomes, which range in size from 30–150 nm, are secreted within the multivesicular endosomes (MVEs), fused with the cell surface, and then released, making them intermediates within the endosomal system [6, 7]. Different from exosomes, microvesicles (50–1000 nm) are secreted directly through ectocytosis, which allows the release of plasma membrane vesicles [8, 9]. The key difference between microvesicles and exosomes is that the intracellular membrane is not involved during the secretion of microvesicles [2]. In contrast, apoptotic bodies are typically larger with a size from 1–5 μm, which are generated during the process of programmed cell death [10]. Various types of EVs participate in the diverse biological processes, such as cell motility [11–13], differentiation [14–16], proliferation [17, 18], apoptosis [10, 19], reprogramming [20–22], and immunity [23, 24]. The connection between EVs and those biological processes has led to their clinical potential [25]. For example, EVs can be targeted to prevent harmful effects associated with EV-mediated communication, which could help to treat diseases such as cancer [26], cardiovascular diseases [27, 28], neurological disorders [29, 30], and immune diseases [31, 32]. Additionally, EVs can serve as biomarkers for lipid biopsy, aiding in the diagnosis and monitoring of challenging diseases [1–4]. Furthermore, EVs can also effectively deliver cargo, including drugs and nucleic acids, to targeted tissues or organs because of their transportation capability and ability to cross biological barriers, such as the blood–brain barrier [2]. Another example of its clinical application is that EVs can be used in drug delivery systems to carry bio/chemical drugs into the pathological tissue as they possess several natural advantages, such as natural barriers traversing capacity, intrinsic cell targeting properties, and stability in the circulation [33–37] (Fig. 1).

Understanding the regulatory mechanisms of EVs in intercellular communication is crucial for their clinical applications [38–46]. This is because most of the functions of EVs towards cells depend on how EVs interact with the recipient cells [38, 47–49]. For instance, Zheng et al. demonstrated that inhibition of exosome uptake interrupted the communication between multiple myeloma cells and bone marrow stromal cell. Thus it is a potential adjunctive strategy for multiple myeloma treatment [50]. Knowing how EVs communicates with cell helps us better understand how they function and then helps the development of the clinical application of EVs. Cellular communication based on EVs includes cell targeting, the release of cargos of EVs, including DNA, RNA, protein, and lipid via cellular uptake and fusion, and transmission of the signal [51–56]. However, those steps are unnecessary for all the interactions [2, 57–59]. For example, exosomes can influence the phenotype of the targeting cell by releasing the cargo and transmitting the signal to recipient cells after the cellular uptake. Nevertheless, an immune reaction is a common function of EVs, and cellular uptake is not necessary. Studies have shown that exosomes with major histocompatibility complex-peptide could directly activate immune cells [60].

**Fig. 2:**
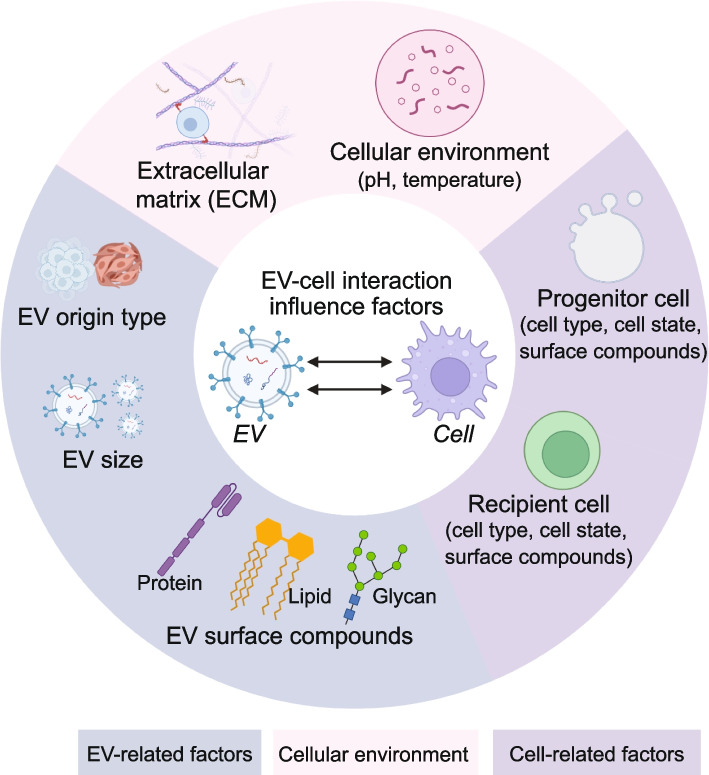
The influence factors for the EV-cellular interaction. The influence factors include the surfaced characteristic molecules related to EV, progenitor cell and recipient cell; and the cellular environments of EVs. The characteristic surface molecules include protein, lipid, and glycan, while the cellular environments include temperature, pH and ECM

There is excessive diversity of the intercellular communication mechanisms based on EVs (Fig. 2), which is related to (i) EV-associated factors including the EV origin type [61], size [62], surface compounds, such as protein [53, 55, 59], lipid [63] and glycan [64]; (ii) Cellular environment around the cell, including extracellular matrix (ECM) [65], and various microenvironmental factors (pH [66], temperature [67]); (iii) Cell-related factors including cell type, cell state and surface compounds of progenitor and receipt cell [63, 68, 69].

Here, we review current knowledge that governs the regulatory mechanisms of EVs in intercellular communication. We discussed the various factors that influence EV-related communication, including targeting, binding, and uptake. Some of the research results contradict others due to the different research strategy, which is also discussed in this review.

## Targeting, binding, and uptake of EVs

### Targeting

There are two questions related to the targeting orientation of exosomes toward the cell, including (1) Are there any signal compounds that determine the direction of exosomes in the biological environment? There is no clear evidence showing that any signal compounds lead to the movement of exosomes toward any organ or a specific type of cell [55, 70]. Recent studies have shown that exosomes can be non-selectively incorporated into various types of recipient cells [71]. However, another study shows that there is a possibility that cellular targeting is based on signaling molecules such as morphogen, with a source-sink mechanism being suggested [72]. Indeed, a biodistribution study shows that red blood cell-derived EVs predominantly target the liver and bone [73]. Also, melanoma-derived EVs were mainly taken up by the lungs and spleen [72]. As well as organ targeting, several studies have shown that EVs have a natural targeting capability based on donor cells [74]. For example, Sharif et al. have found that mesenchymal stem cells (MSCs) can deliver miRNAs into glioblastoma (GBM) cells through EVs [75]. This may be because EVs express specific lipid and cellular adhesion molecules related to the progenitor cell, which has the specificity for certain receptor cells. This cell-type-specificity targeting may be related to receptor-ligand binding, which leads to the second question. (2) Whether the cell/tissue/organ targeting is associated with molecular signaling or the selective uptake of the recipient cells. EV uptake mainly occurs when the EVs and the receptor cells share the suitable ligand and receptor [55]. Cell specificity targeting is likely to be determined by the exclusive interactions between ligands enriched at the surface of EVs and receptors at the plasma membrane of the recipient cells. There is clear evidence of the binding specificity because of the receptor-ligand interaction, which shows that the targeting orientation may be related to the binding specificity [54, 76]. For example, one subtype exosome from neuroblastoma cells specifically targets them to neurons, because of the presence of amyloid precursor protein while another exosome subtype binds both neurons and glial cells [77]. Targeting and distribution are essential for drug delivery design; they are determined by cell source, route of administration, circulation time, as well as the uptake selection related to EV-cell interaction [70, 78, 79]. EV-based drug delivery systems can be engineered for enhanced specificity in targeting specific cell types, through either genetic manipulation or chemical modification [36]. Genetic engineering involves fusing ligands or homing peptides with transmembrane proteins expressed on the surface of exosomes [80]. To achieve this, donor cells are transfected with plasmids encoding the fusion proteins, which results in the secretion of engineered exosomes displaying targeting ligands on their surface. The surface protein LAMP-2B is currently the most commonly used for displaying targeting motifs [81]. Another way to enhance targeting is through chemical modification, which can be accomplished through conjugation reactions or lipid assembly [36].

### Binding

EVs bind to target cells via specific surface receptors, triggering various intracellular signaling pathways that can affect cellular behavior and function. The binding of EVs to target cells is a complex process that involves several factors, including the composition of the EV membrane, such as protein, lipid, and glycan (Table 1). Understanding the mechanisms of EV binding is critical for developing new strategies to manipulate intercellular communication and improve disease diagnosis and treatment.

**Table 1:** The surface biomolecules of EV involved in intercellular communications

Category	Sub-category	Example	Roles	Ref
*Protein*	Tetraspanin	CD9	EV uptake	[82]
		CD63	EV uptake	[82]
		CD81	EV uptake	[82]
		CD82	EV uptake	[82]
	Lectin	transmembrane C-type lectins	EV uptake	[63]
		transmembrane Siglecs	EV uptake	[63]
		cytosolic galectins	EV uptake	[63]
	Integrin	α6β4, α6β1	EV uptake	[83]
		αvβ5	EV uptake	[83]
	Scaffold protein	ALIX	EV biogenesis	[84]
		Syntenin	EV biogenesis	[85]
	Antigen-presenting protein	MHC class I	Immune response	[86]
		MHC class II	Immune response	[86]
*Lipid*	Glycerophospholipids	phosphatidylserine	EV signaling and uptake	[87]
	Sphingolipids	Ceramide	EV signaling	[88]
	Cholesterol	Cholesterol	EV secretion	[89]
*Glycan*	Proteoglycan	HSPG	EV uptake	[90, 91]

#### Protein

Proteins have been recognized as essential participants which significantly contribute to receptor-ligand recognition through protein–protein, protein-lipid, and protein-glycan interaction [52–55, 63, 71, 92–94]. Those proteins involved in the binding can be generally classified into several groups, such as tetraspanins, lectins, integrins and scaffold proteins [52, 68, 95, 96] (Fig. 2).

Tetraspanins are also termed as four transmembrane crosslinked proteins, which are vastly abundant on the surface of EVs [82]. CD9, CD63, CD81 and CD82 are broadly distributed tetraspanins which are well-established markers of EVs [82, 97]. Apart from acting as biomarkers, tetraspanins play an essential role in EV docking and uptake by interacting with integrins and adhesion receptors [82]. Tetraspanin-enriched microdomains (TEM) on the surface of EVs gather tetraspanins, adhesion molecules, and transmembrane receptor proteins, and these primary raft-like structures interact with a large variety of transmembrane and cytosolic signaling proteins [98, 99]. Adhesion molecules, such as intracellular adhesion molecule (ICAM), are commonly present in TEMs and are involved in the binding of exosomes to the target cells, particularly immune cells [99, 100]. Tetraspanin on the surface of EVs contributes to target cell selection because of the selective binding interactions between EVs and cell [99]. For instance, a study showed that the in vivo uptake of the target cell is significantly selected owing to the differences between the exosomes expressing different tetraspanins [101]. The selectivity of target cells for EVs expressing different tetraspanins can regulate cancer progression and metastasis and serve as a tailoring factor for engineered EVs in drug delivery [99].

Lectins are a large protein family that facilitate cell-to-cell communication by recognizing and binding glycan moieties [96]. Choi et al. showed that the interactions between lectin and glycan have the inherent potential to capture exosomes derived from cancer [102]. The lectin proteins can be grouped into three groups: transmembrane C-type lectins and selectins, transmembrane Siglecs, and cytosolic galectins [63]. A large number of lectins have various binding specificities that can partially overlap with each other and some similar members can function complementally in the absence of another. Some lectins are involved in immunity based on EV signaling [63]. L-selectin is one type of selectin that localizes on the surface and mediates the cell adhesion for immune cells such as granulocytes, monocytes, lymphocytes, and leucocytes [103]. Siglec-2 (CD22) is another important transmembrane protein of lectins, which prevents autoimmunity by inhibiting signaling related to B-cell receptors (BCR) [99].

The integrin family of proteins include a diversity of heterodimeric membrane cell adhesion proteins, and they consist of α and β subunits as well as an integral component of integrin-adhesion complexes [104, 105]. Integrins regulate several biological processes, such as cell proliferation, differentiation, apoptosis, and migration, by mediating signaling pathways [93, 106]. The integrins are involved in both cell–cell and cell-EV interactions, through invasion [53, 93]. Furthermore, the exosome proteomics study revealed that exosomal integrins α6β4 and α6β1 have been linked to lung metastasis, while exosomal integrin αvβ5 is associated with liver metastasis. This study provided a potential therapeutic strategy since targeting the integrins α6β4 and αvβ5 decreased exosome uptake and prevented lung and liver metastasis, respectively [83].

Scaffold proteins, such as ALIX and Syntenin, are also essential in EV-cell interactions, as they are critical mediators in exosome biogenesis [107]. It has been discovered that ALIX plays a direct role in facilitating the loading of PD-L1 onto exosomes from the endosomal lumen [108]. Cells with ALIX deficiency exhibit decreased levels of PD-L1 on exosomes, but retain the expression of PD-L1 on the surface of tumor cells [84]. In addition, the biogenesis of EVs can be influenced by other proteins, such as heparinase, which can interfere with the interaction between syntenin and ALIX [85, 109].

#### Lipid

Lipids are essential molecules in the EVs’ membrane and play a crucial role in the interaction between EVs and cells [110]. The membranous lipids can be classified into several groups based on the different structures of lipids, such as glycerophospholipids, sphingolipids, and cholesterol. Each group of lipids has several variations related to carbon atoms and double bonds [89]. Lipids may organize, transmit “mobile rafts”, spread the signals, and then activate cell signaling pathways involved in oncogenesis and metastasis [111]. Surface phosphatidylserine is one example of glycerophospholipids is involved in cellular communication [87]. The interaction between phosphatidylserine and its receptor Tim4 is Ca^2+^-coordinated which has been thoroughly investigated. In addition to Tim4, there are many other receptors have been explored in the past years, such as the advanced glycation end products, RAGE [112], brain-specific angiogenesis inhibitor 1, Bai-1 [68], and stabilin-2 [113]. Moreover, phosphatidylserine receptor stabilin-2 and Bai-1 perform a key function in the rapid clearance of cell corpses [112], via recognizing phosphatidylserine in aged red blood cells and apoptotic cells and mediating the engulfment [88, 114]. Similarly, phosphatidylserine is indirectly recognized by the growth arrest-specific protein 6, Gas6. The phosphatidylserine-Gas6 complex activates the MER tyrosine kinases on the surface of macrophages which triggers the EV uptake and causes an anti-inflammatory phenotype [115]. Ceramide is a critical sphingolipids enriched on the surface of EVs, and it is involved in the function of mobile rafts and affects the cell signaling pathways [88].

#### Glycans

Glycans play a crucial role in binding of EVs to cells through the sugar-sugar and sugar–protein reactions [116, 117]. Proteoglycan (PG) is a glycosylated protein whose main structure is glycosaminoglycan [118, 119]. PG is one of the most studied glycan structures with polyamines and domains of basic amino acids present, which has the polyanionic charge because of their glycosaminoglycan chains [118]. And PG is involved in a wide variety of binding reactions with polybasic ligands, such as polyamines, nucleic acid-peptide complexes, cationic lipids, viral capsid proteins, and apolipoproteins [64]. Inhibiting either proteoglycans or its receptors like lectin would reduce the EV uptake [56, 120].

One example of PG is heparan sulfate proteoglycan (HSPG), such as the plasma membrane protein of GPI-linked glypican family and the transmembrane protein of syndecan family [91]. HSPG plays a crucial role in EV-mediated communication in the tumor microenvironment as it is necessary for efficient growing factor signaling and serves as the initial attachment sites and true internalizing receptors of macromolecular ligands [90, 91]. In addition to tumors, HSPG is also involved in other disease initiations and development. For example, changes in proteoglycans induced by abnormal insulin and fatty acids exposure in hepatic cells are associated with insulin resistance-associated dyslipidemia [69]. As well as proteoglycans, glycolipids and glycoproteins also widely participate in the binding interaction for EV-mediated uptake. Furthermore, glycosylated sialic acid- and mannose-containing glycoproteins, are also glycan structures involved in the EV binding interaction and they significantly influence the EV uptake [121].

As glycans are key players in the EV binding process of EV uptake, the engineering of glycan can serve EV-based therapy in various ways by controlling the EV uptake [94]. One of the glycan-based strategies for EV-related therapy is drug delivery designing [56]. The modification of the glycosylation of lipids and protein on the surface of the engineered EVs would alter the distribution of EV by influencing the EV uptake of the target cell [33, 34, 122]. Another therapeutic approach is to directly target EV-HSPG interactions, which further control EV uptake for cancer treatment [64]. For example, heparan sulfate exposed on the plasma membrane acts as an EV receptor, regulating the EV uptake of cancer cells [123].

#### The size of EVs

The size of an EV is related to its heterogeneity, which is influenced by its origin, type, and characteristics [2, 5]. For example, larger EVs are typically associated with microvesicles while smaller EVs are often related to exosomes [2]. This heterogeneity can affect their cellular interactions. Also, the size of EVs has been linked to cell targeting and uptake rates. Caponnetto et al. have proved that the smaller engineered EVs are associated with increased cellular motility which then accelerates the EV uptake [124]. This means that smaller EV leads to a more effective delivery of their cargo and signals, which would improve the drug delivery efficacy. Besides, Yang et al. showed that smaller exosomes can target tumor tissue via enhanced permeability and retention [125]. Furthermore, larger EVs may increase the EV circulation time in the blood and retard their clearance, which would improve the EV uptake by recipient cells [92].

#### Cellular environment

EVs generally travel long distances to communicate, and the cellular environment is a mediator between EVs and cells, which have a certain level of influence in EV-cell interaction [126]. The cellular environment includes ECM, as well as the microenvironment, such as the condition of pH, temperature, oxidative/hypoxic state, and other factors. The modulation of these conditions may influence EV uptake. For example, variations in the stiffness of the ECM can modulate EV uptake [126], and the mechanical properties of the matrix can regulate EV transport by crosslinking with water permeation [127]. Moreover, ECM affects the astrocytic EVs in wound recovery, as EVs from the ECM-exposed astrocytes exhibit accelerated rates of wound recovery [66].

In addition, cellular environment condition also influences EV-medicated functions. For instance, oxidative stress affects the protein content of exosomes secreted from amnion-epithelial, which releases inflammatory mediators that lead to the inflammation reaction [94, 128]. Similarly, the hypoxic state also affects exosome miRNA and protein contents [129, 130]. Furthermore, pH significantly influences EV uptake related to the individual EV-cell interaction [131, 132]. For example, a low pH condition can benefit tumor malignancy by potentially influencing exosome release and uptake by cancer cells [131]. As well as pH, temperature shows a significant influence on the cellular uptake of EVs. Cellular uptake of EVs is strongly inhibited at low temperatures, indicating an energy-dependent process [51, 133]. Radiation can also be considered as another uncertainty from the cell environment influencing EV-cell communication. For instance, Jabbari et al. showed that ionizing radiation can increase the activity of exosomal secretory pathway in breast cancer cells, which is a promising method for the resistance against radiotherapy [134].

#### Progenitor cell and recipient cell

EV-cell interaction is associated with the progenitor cell as the composition and characteristics of the EVs can be influenced by the EV progenitor cell. For example, a lipidomic study has proved that EVs from PC-3 prostate cancer cells contain a significant enrichment of certain glycolipids on the surface and these EVs contain more than eight times higher content of lipids/protein ratio than the parent cells [135]. Such differences in composition can affect EV-cell interaction. Sancho-Albero et al. showed that the engineered exosomes originated from 3 different human carcinoma cell lines exhibit variable targeting efficiency towards different cells, which also influences the efficiency of delivering therapeutic nanoparticles to their intended target cells [136]. Moreover, the characteristics of EVs can also be influenced by the state of the cells that secrete exosomes, which further influence cellular binding and uptake. For example, macrophages and mature dendritic cells have been shown to uptake more EVs than monocytes or immature dendritic cells [137]. In addition, the type of the recipient cell can also influence the interaction with EVs, owing to the involvement of specific ligand and receptors. Horibe et al. showed that the different recipient cells mediate exosome uptake through different mechanisms, and the endocytosis can be inhibited by different inhibitors depending on the recipient cells [71].

### Uptake route

**Fig. 3:**
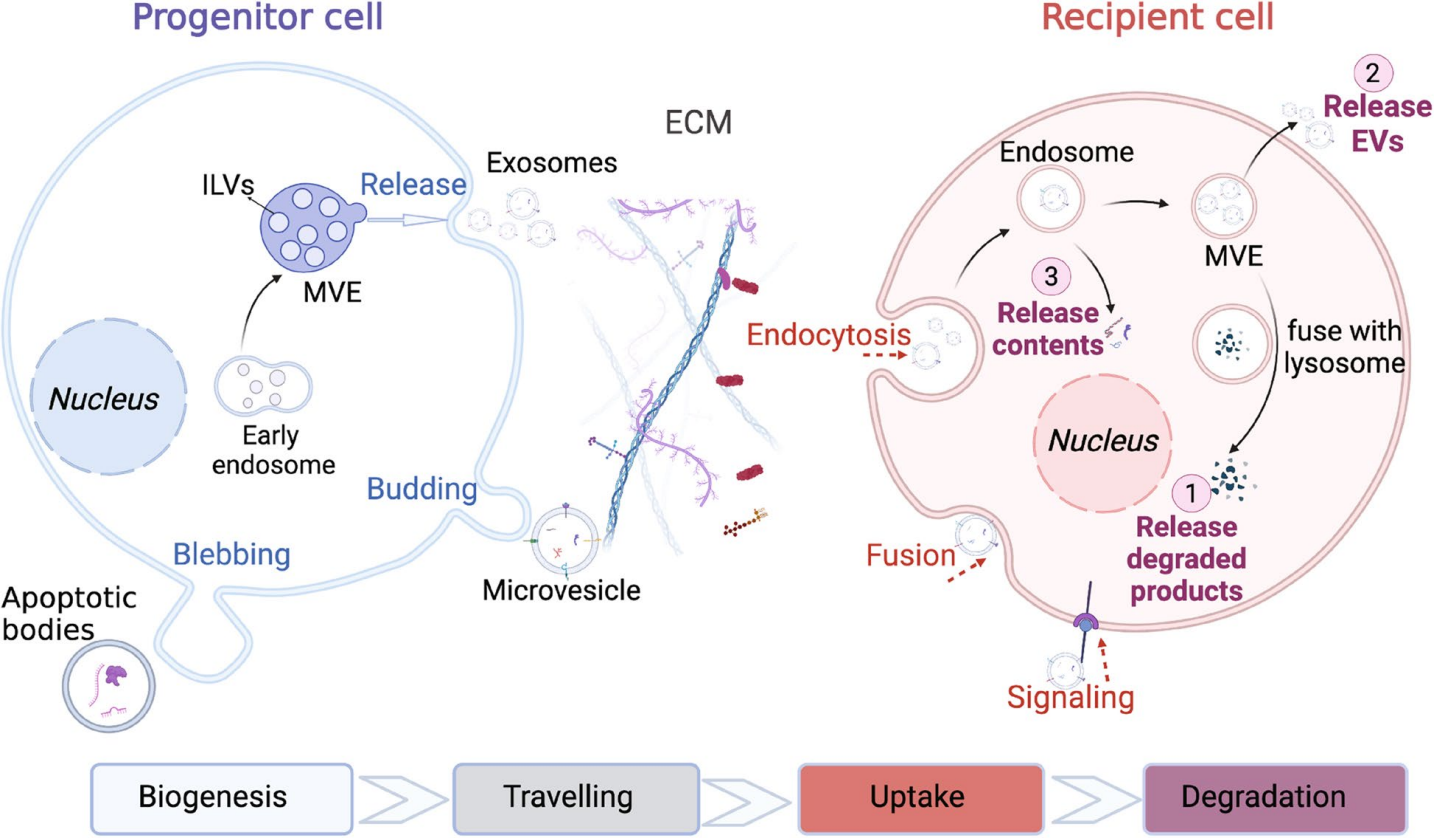
The biogenesis, travelling, uptake and degradation of EVs. EVs biogenesis occurs in progenitor cells. Then various EVs are secreted by progenitor cells, such as exosomes, microvesicles and apoptotic bodies. Exosomes are secreted within the MVEs. Microvesicles are secreted by directly budding through the plasma membrane and apoptotic bodies are released via membrane blebbing. After traveling through the ECM, EVs may influence the cell phenotype by endocytosis, fusion, and signaling. Subsequently, EVs would go through their metabolism in the recipient cell via three possible pathways including degradation by the lysosome, being released outside of the recipient cell directly, and contents release into the cytoplasm of the recipient cell. (Abbreviation: ECM, extracellular matrix; MVE, multivesicular vesicle; ILVs, intraluminal vesicles)

After binding to the surface of the recipient cells, EVs can influence the cell phenotype through various uptake mechanisms, such as endocytosis, fusion and signaling (Fig. 3). Endocytosis is one of the common uptake ways for EVs to transmit signals, and it occurs through different pathways, including clathrin-dependent pathways, caveolin-mediated uptake, macropinocytosis, phagocytosis, and lipid raft-mediated internalization [3, 138]. The internalization pathways of EVs are similar to the internalization pathways of most extracellular particles, including viruses [139]. The uptake pathways of EVs are influenced by several factors, including the characteristics of EVs (surface protein, lipid, glycan and size), recipient cell, and the cell environment (e.g., pH and temperature). For example, CD47 on the surface of the exosomes has a strong inhibitory effect on the internalization of EVs to monocytes via phagocytosis [140]. The lipid composition of both EVs and the recipient cells also largely influences the uptake mechanism, as lipid rafts contribute significantly to EV internalization [51]. Lipid rafts are lipid microdomains enriched in cholesterol, glycosphingolipids, and glycosyl-phosphatidylinositol GPI-anchored proteins, which are related to several types of endocytosis, including caveolin-dependent endocytosis, micropinocytosis [141]. Fusion is another internalization uptake pathway, where the EV membrane directly fuses with the cell plasma membrane [55]. Bonsergent developed a cell-based assay using a fluorescent tag to demonstrate the involvement of membrane fusion [142].

The specific uptake pathway can be elucidated using inhibitors that prevent the associated receptor-ligand interactions [57, 143]. For instance, clathrin-mediated endocytosis requires the assembly of clathrin-coated vesicles which contain a diversity of receptors and their ligands [144]. The molecular impact of the assembly process can intermediate endocytosis. Chlorpromazine is an inhibitor that can prevent the formation of clathrin-coated pits and decreases the uptake of EVs by ovarian cancer cells Similarly, caveolin-1 is a pre-protein for the caveolae-dependent pathway and the oligomerization of caveolins intervenes the formation of caveolin-rich rafts in the plasma membrane [145]. The increased levels of caveolin scaffolding domains activity promote the caveolae-dependent pathway [77, 146], while the inhibitor dynasore has the opposite effect. Notably, EV uptake may occur through a combination of pathways or another mechanism if the preferred pathway is blocked [145]. This can make it challenging to explore the internalization mechanism using a given inhibitor [147].

In addition to internalization, EVs can trigger intracellular signaling pathways through direct interaction with the surface receptors or ligands of target cells. EV signaling can affect the cell phenotype via the membrane-bound morphogens, such as Wnt [148–150], and the Notch ligand DII4 [151]. EV signaling can also affect the cell motility, migration, and invasiveness of cancer cells. For example, breast cancer cell metastasis can be promoted through exosome-mediated paracrine Wnt10b [152] and autocrine Wnt-PCP signaling [148]. Furthermore, immune regulation is another way of transmitting signaling without endocytosis. T cells (CD8 + and CD4 +) can be directly stimulated by EVs which carry surface antigen-presenting molecules, including MHC class I and MHC class II complexes [86].

### Intracellular trafficking and signal transmission

After binding and uptake to recipient cell, EVs follow the endocytic pathway within the cell. There are several pathways of EV degradation [7]. The most common one is to reach MVEs and then be degraded by lysosome [1]. In this case, the contents are degraded as well, which is correlated to the waste management function of EVs [7]. Alternatively, EVs may not be degraded within the cell and instead be released directly. A third way is that the inter nalized EVs escape from digestion and then release their contents into the cytoplasm of the recipient cell [2]. Although this pathway has a relatively low chance of occurrence, it is noteworthy due to the potential impact on the recipient cells. With the contents including protein, lipid, DNA and RNA being released into the cell, those content can then regulate the recipient cell phenotype and function. One of the significant contents founds in EVs is RNA, particularly miRNAs [153, 154]. miRNAs are the most known mediators of cell–cell communication based on EVs [154]. One of the examples is miR-193b, which is secreted in the central nervous system and is associated with the expression of amyloid precursor protein in neuronal cells. Its analysis related to cellular communication provides a promising therapy and biomarker for Alzheimer disease [155]. Moreover, protein and lipid cargoes in EVs can also trigger several reactions and activities in the recipient cell after internalization. It has been shown that protein cargoes can contribute to immune response regulation [156, 157] while lipid species contribute to the regulation of bioactive lipid species [158]. For example, chemokine receptor CCR5 can be released by macrovesicle, which regulates the human cellular immunodeficiency against virus 1 infection [159]. Taking advantage of the natural transportation property of the EVs, they can be used for drug delivery of various therapeutic agents, including chemotherapy [35], neurological disorder medicine [160] and nucleic acids [161].

## Conclusions

EVs play a crucial role in transmitting information between cells and influencing the behavior and function of recipient cells. Understanding of regulatory mechanisms of EVs in intercellular communication is critical for their clinical application. Although significant progress has been made in understanding the factors that govern intercellular communication through EVs, including the molecular properties of EVs, the cellular environment, and the recipient cell, there is still much to be learned about the regulations that control EV targeting and cellular uptake. Furthermore, the mechanisms of cargo release and intracellular trafficking within the recipient cell are not yet fully understood owing to the small size of EVs and the lack of related imaging technology. Besides, more research is needed to unravel the distinct mechanisms of various EV subpopulations in cellular uptake, as it is challenging to isolate, identify, and characterize different EV subpopulations. Despite these challenges, the clinical potential of EVs is a major driving force for ongoing research in this field. With further research and technological advancements, we can expect to gain a deeper understanding of the mechanisms of EV-mediated intercellular communication, which will ultimately help us develop new therapeutic strategies for treating various diseases.

## Supplementary Information

**Additional file 1.**

## Data Availability

The data and materials are available upon the request.
